# Scalp nodules as the first presentation of prostate cancer: A CARE-compliant article

**DOI:** 10.1097/MD.0000000000036570

**Published:** 2023-12-15

**Authors:** Tae Hoon Oh, Hun Soo Kim, Seung Chol Park

**Affiliations:** a Department of Urology, Wonkwang University Hospital, Institute of Wonkwang Medical Science, Iksan, Korea; b Department of Pathology, Wonkwang University Hospital, Institute of Wonkwang Medical Science, Iksan, Korea.

**Keywords:** prostate cancer, skin

## Abstract

**Rationale:**

Bones are the most common site of prostate cancer metastasis. Other common sites of metastases include the distant lymph nodes, liver, thorax, brain, and digestive system. However, cutaneous metastases from prostate cancer are extremely rare.

**Patient concerns:**

We present a case of a 61-year-old man with scalp nodules without any cancer history.

**Diagnosis:**

The patient was diagnosed with metastatic prostate adenocarcinoma through an incisional biopsy for his scalp nodules. The patient presented with a serum prostate-specific antigen level of 10.2 ng/mL; imaging examinations revealed extraprostatic extension, lymph node involvement, and multiple bone metastases.

**Intervention:**

The patient was treated with androgen deprivation therapy with leuprolide acetate (7.5 mg every month) and abiraterone acetate (1000 mg daily).

**Outcomes:**

The scalp metastases resolved without adverse effects, and the serum prostate-specific antigen level decreased to 0.02 ng/mL.

**Lessons:**

Cutaneous metastasis, especially scalp metastasis from prostate cancer, is extremely rare. If there is a rash or nodule on the skin, it is necessary to evaluate it carefully and to confirm it through a biopsy.

## 1. Introduction

Prostate cancer is the third most common cancer in Korean men, with an estimated number of 22,837 new cases in 2023.^[1]^ Bones are the most common site of prostate cancer metastasis. Other common sites of metastases include the distant lymph nodes, liver, thorax, brain, and digestive system.^[2]^ Cutaneous metastases from prostate cancer are extremely rare, with incidence rate of 0.62% to 0.7%, as established through meta-analyses.^[3,4]^ Cutaneous metastases from prostate cancer typically occur in the inguinal region, penis, abdomen, head and neck, chest, extremities, and back and are rare on the scalp.^[5]^ Cutaneous metastases from prostate cancer usually appear in the later stages of the disease. Scalp nodules are an extremely rare manifestation of prostate cancer. Herein, we reported a case of a 61-year-old man who presented with scalp metastases as the first manifestation of prostate cancer.

## 2. Case presentation

**Figure 1.**
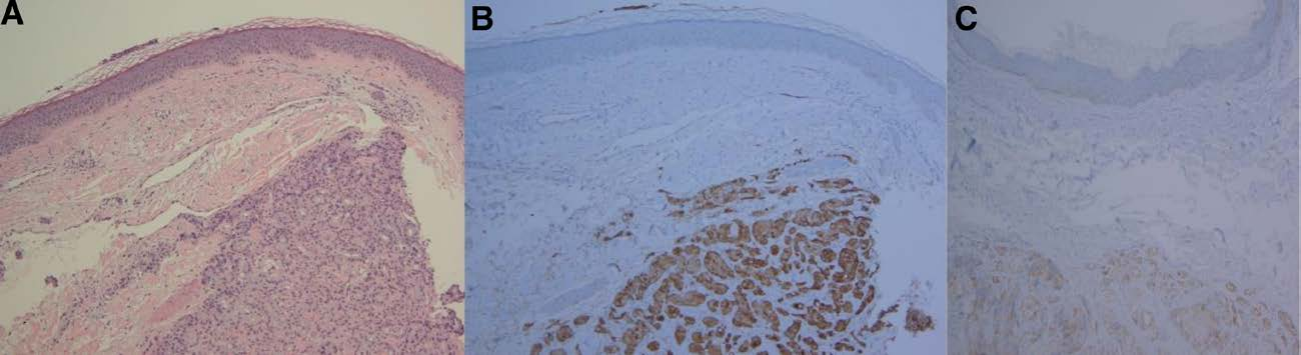
Sheet-like infiltration of small crowded acini with round monomorphic nuclei in the reticular dermis. The morphologic architecture spectrum consists of well-formed (Gleason grade 3) to complicated (Gleason grade 4) glandular proliferation (H&E ×100). The atypical glands exhibit strong serum prostate-specific antigen (PSA) and moderate alpha-methylacyl-CoA racemase (P504S) immunohistochemical reactivity (PSA, P504S ×100).

A 61-year-old Korean man was admitted with multiple skin-colored papules and nodules on his scalp. Five months ago, he noticed the development of 3 papules on the scalp. Over time, these papules exhibited gradual enlargement and a change in color to red, while 3 additional nodules emerged in different locations on the scalp. The patient visited a private dermatologic clinic seeking medical attention for his nodules. He was recommended to undergo a biopsy and diagnostic workup and was transferred to our hospital’s dermatology department. The patient underwent an incisional biopsy of one of several nodules, which revealed prostate adenocarcinoma (Gleason grade 2) with positive serum prostate-specific antigen (PSA) and P504S staining in immunohistochemical analysis (Fig. 1). He was referred to our department for further evaluation and treatment of prostate cancer.

**Figure 2.**
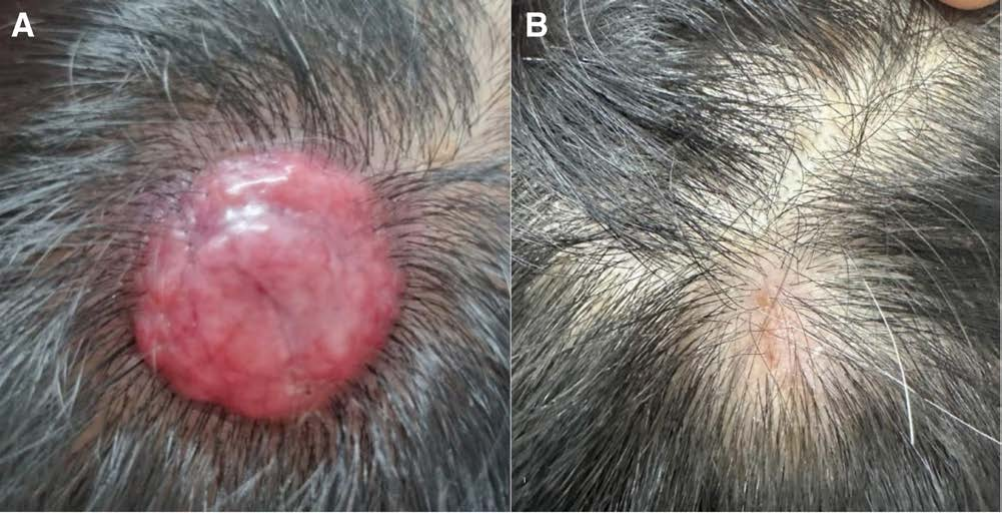
(A) Multiple red, non-tender, dome-shaped papules with diameter 0.5 to 2 cm are present on the scalp. (B) The scalp lesions disappear after 3 months of treatment.

**Figure 3.**
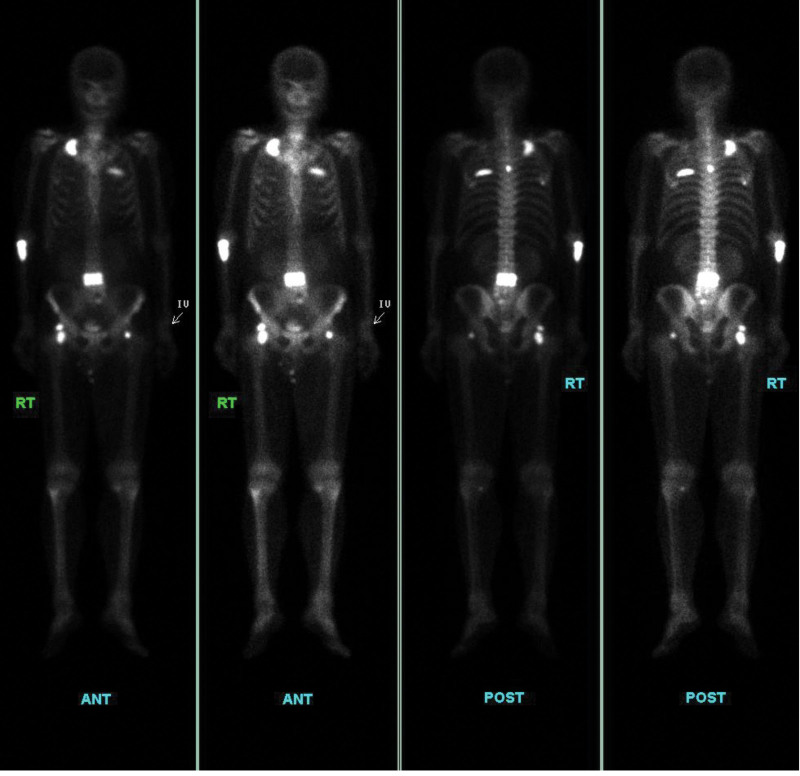
A whole-body bone scintigraphy reveals extensive bony metastases in the vertebrae, multiple ribs, right proximal radius, and bilateral proximal femurs.

On examination, multiple red, non-tender, dome-shaped papules and nodules of diameter 0.5 to 2 cm were present on the scalp (Fig. 2A). The serum PSA level was 10.2 ng/mL, and the alkaline phosphatase level was 150 U/L (40–129). Imaging examinations, including magnetic resonance imaging of the prostate and computed tomography scans, revealed extraprostatic extension, seminal vesicle invasion, lymph node involvement, and multiple bone metastases (Fig. 3). The patient was treated with androgen deprivation therapy using leuprolide acetate (7.5 mg monthly) and abiraterone acetate (1000 mg daily). He responded well to the treatment, experiencing only mild lower urinary tract symptoms. Imaging investigations performed 3 months later showed a partial response, with the scalp lesions disappearing and the serum PSA levels decreasing to 0.02 ng/mL (Fig. 2B).

This study was approved by the Wonkwang University Hospital Institutional Review Board (WKUH 2023-05-013). The patient has provided written informed consent for the publication of the case.

## 3. Discussion

Cutaneous metastases originating from genitourinary malignancies are uncommon, occurring in 1% of patients with advanced disease. The incidence of cutaneous metastases from the kidney, bladder, prostate, and testes is 3.4%, 0.84%, 0.36%, and 0.4%, respectively. The most common presentation includes an infiltrated plaque or nodules. Cutaneous metastases from genitourinary malignancies are associated with poor prognosis, with the median disease-specific survival of <8 months.^[6]^

The most common metastatic sites from prostate cancer are the bones (84%), followed by the distant lymph nodes (10.6%), liver (10.2%), thorax (9.1%), brain (3.1%), and digestive system (2.7%).^[2]^ Cutaneous metastases from prostate cancer are extremely rare, with a prevalence rate of 0.62% to 0.7% established through meta-analyses.^[3,4]^ The sites of metastases can vary, with the most common being inguinal lesions (scrotum, penis, pubis) and the second most common being chest lesions. Many distant metastases involve other cutaneous disease sites, such as the groin, face, and back.^[7]^ Scalp and face metastases are very rare. Other rare sites of metastasis are umbilical nodules and laparoscopic scars. The gross appearance of cutaneous metastases from prostate cancer can vary widely, ranging from multiple rubbery nodules or plaques to single nodules or even presenting as edema or a nonspecific rash. These lesions may be asymptomatic, painful, or ulcerated, and their appearance can resemble other conditions, such as herpes zoster, basal cell carcinoma, angiosarcoma, cellulitis, pyoderma gangrenosum, mammary Paget disease, telangiectasia, or sebaceous cysts.^[8]^ Here in, we reported an extremely rare presentation of scalp metastases as the initial symptom of prostate cancer without a previous history of malignancy. Previous studies have also reported scalp metastases from prostate cancer, such as the study by Spitz et al,^[9]^ where 2 out of 59 (3%) scalp lesions were observed to have metastasized from prostate cancer as revealed through a series of fine needle aspiration.

Skin biopsy and immunohistochemical staining can confirm whether the presenting lesion is primary or secondary to metastasis. Staining of PSA, prostate acid phosphatase, and prostate-specific membrane antigen usually display high specificity to prostate epithelial cells.^[10]^ In this case, the morphological architecture spectrum consisted of a well-formed (Gleason pattern 3) to complicated (Gleason pattern 4) glandular proliferation, with the atypical glands exhibiting strong PSA and moderate alpha-methylacyl-CoA racemase in immunohistochemical reactivity.

Most cutaneous metastases are often discovered in advanced-stage diseases, and scalp metastases from prostate cancer most commonly occur during treatment, follow-up after diagnosis, or as the first symptom of castration-resistant prostate cancer. When cutaneous metastases are identified, they often coexist with other metastases in other organs, including bone metastases. Therapeutic options for cutaneous metastases are primarily palliative and include excision, radiation, and intralesional chemotherapy.^[4]^ Because of the limited treatment options available, a multidisciplinary approach is needed.

This case was a very rare case of scalp metastases serving as the first symptom of prostate cancer with lymph node metastases and bone metastases. Cutaneous metastases are associated with widespread disease and poor prognosis. In our case, the scalp lesions resolved after treatment with luteinizing hormone-releasing hormone agonist plus abiraterone acetate for metastatic hormone-sensitive prostate cancer.

## 4. Conclusions

Cutaneous metastasis, especially scalp metastasis from prostate cancer, is extremely rare. Most cutaneous metastases occur when the disease is advanced; however, in this case, scalp metastases occurred as the first symptom of prostate cancer. Although the overall rate of cutaneous metastases is low, a skin lesion may represent an undiagnosed metastasis, highlighting the importance of careful evaluation and confirmation through a biopsy.

## Acknowledgments

This paper was supported by Wonkwang University in 2021. We would like to thank Editage (www.editage.co.kr) for English language editing.

## Author contributions

**Conceptualization:** Tae Hoon Oh, Hun Soo Kim, Seung Chol Park.

**Data curation:** Hun Soo Kim, Seung Chol Park.

**Formal analysis:** Seung Chol Park.

**Funding acquisition:** Seung Chol Park.

**Investigation:** Tae Hoon OH, Seung Chol Park.

**Methodology:** Hun Soo Kim, Seung Chol Park.

**Project administration:** Seung Chol Park.

**Resources:** Hun Soo Kim, Seung Chol Park.

**Software:** Seung Chol Park.

**Supervision:** Seung Chol Park.

**Validation:** Seung Chol Park.

**Visualization:** Hun Soo Kim, Seung Chol Park.

**Writing – original draft:** Tae Hoon OH, Seung Chol Park.

**Writing – review & editing:** Seung Chol Park.
